# Ferulic acid alleviates lipotoxicity-induced hepatocellular death through the SIRT1-regulated autophagy pathway and independently of AMPK and Akt in AML-12 hepatocytes

**DOI:** 10.1186/s12986-021-00540-9

**Published:** 2021-01-19

**Authors:** Tiantian Xu, Qing Song, Li Zhou, Wenwen Yang, Xiangyao Wu, Qianyu Qian, Hui Chai, Qiang Han, Hongzhi Pan, Xiaobing Dou, Songtao Li

**Affiliations:** 1 College of Basic Medicine and Public Health, Zhejiang Chinese Medical University, Hangzhou, 310053 China; 2 College of Life Science, Zhejiang Chinese Medical University, Hangzhou, 310053 China; 3 Molecular Medicine Institute, Zhejiang Chinese Medical University, Hangzhou, 310053 China; 4 The First Affiliated Hospital of Zhejiang Chinese Medical University, Zhejiang Chinese Medical University, Hangzhou, 310053 China; 5 Collaborative Research Center, Shanghai University of Medicine and Health Sciences, Shanghai, 201399 China

**Keywords:** Ferulic acid, SIRT1, Autophagy, Lipotoxicity, Metabolic diseases

## Abstract

**Background:**

Lipotoxicity-induced cell death plays a detrimental role in the pathogenesis of metabolic diseases. Ferulic acid, widespread in plant-based food, is a radical scavenger with multiple bioactivities. However, the benefits of ferulic acid against hepatic lipotoxicity are largely unclear. Here, we investigated the protective effect of ferulic acid against palmitate-induced lipotoxicity and clarified its potential mechanisms in AML-12 hepatocytes.

**Methods:**

AML-12 mouse hepatocytes were exposed to palmitate to mimic lipotoxicity. Different doses (25, 50, and 100 μM) of ferulic acid were added 2 h before palmitate treatment. Cell viability was detected by measuring lactate dehydrogenase release, nuclear staining, and the expression of cleaved-caspase-3. Intracellular reactive oxygen species content and mitochondrial membrane potential were analysed by fluorescent probes. The potential mechanisms were explored by molecular biological methods, including Western blotting and quantitative real-time PCR, and were further verified by siRNA interference.

**Results:**

Our data showed that ferulic acid significantly inhibited palmitate-induced cell death, rescued mitochondrial membrane potential, reduced reactive oxygen species accumulation, and decreased inflammatory factor activation, including IL-6 and IL-1beta. Ferulic acid significantly stimulated autophagy in hepatocytes, whereas autophagy suppression blocked the protective effect of ferulic acid against lipotoxicity. Ferulic acid-activated autophagy, which was triggered by SIRT1 upregulation, was mechanistically involved in its anti-lipotoxicity effects. SIRT1 silencing blocked most beneficial changes induced by ferulic acid.

**Conclusions:**

We demonstrated that the phytochemical ferulic acid, which is found in plant-based food, protected against hepatic lipotoxicity, through the SIRT1/autophagy pathway. Increased intake of ferulic acid-enriched food is a potential strategy to prevent and/or improve metabolic diseases with lipotoxicity as a typical pathological feature.

## Introduction

Metabolic diseases, including non-alcoholic fatty liver disease (NAFLD), diabetes mellitus, and obesity, are a worldwide epidemic and commonly feature hyperlipidaemia as a hallmark. Under normal physiological conditions, adipose tissues have a high ability to store excessive fat, and maintain lipid homeostasis; however, in the pathological state of insulin resistance, enhanced lipolysis of adipose tissue leads to elevated heterotopic deposition of free fatty acid (FFA) in non-adipose tissues, such as the liver, skeletal muscle, and pancreas, resulting in cell dysfunction and even cell death, which are termed lipotoxicity and lipoapoptosis, respectively [1]. Because the liver is the central organ of metabolism, hepatic cell death induced by lipotoxicity accelerates the occurrence and development of metabolic diseases. We previously reported that alleviating hepatic lipotoxicity effectively improved high-fat-diet-induced NAFLD in mice [2]. FFAs are chemically divided into saturated fatty acids (SFAs) and unsaturated fatty acids (USFAs). Lipotoxicity-induced cell death is commonly induced by SFAs stimulating oxidative stress and endoplasmic reticulum stress [3, 4], whereas USFAs tend to improve SFA-induced lipotoxicity [5]. Palmitic acid (PA, C16:0) is the most abundant natural SFA in food and the human body and is widely used to induce lipotoxicity for scientific investigations [6, 7].

Autophagy is a highly conserved physiological process by which intracellular components can be degraded and removed for quality control and nutritional purposes. People with lipid metabolism disorders of the liver, such as NAFLD patients, usually suffer from impaired autophagy [2]. Recent studies, including ours, have confirmed that activating autophagy is an effective way to improve lipotoxicity-induced hepatocellular injury in both cultured cells and animal models [2, 8]. Although the mechanisms behind autophagy-regulated lipotoxicity are not fully illustrated, autophagy activation helps to eliminate damaged organelles, such as mitochondria and the endoplasmic reticulum, which are eliminated in processes termed mitophagy and reticulophagy, respectively; moreover, autophagy is involved in protecting against lipotoxicity by improving oxidative stress and endoplasmic reticulum stress [9, 10]. Additionally, autophagy degrades intracellularly accumulated triglycerides in a process termed lipophagy [11] and, in turn, alleviates obesity-associated metabolic disorders in the liver [12].

Several mechanisms have been implicated in the regulation of autophagy. Among these, adenosine monophosphate-activated protein kinase (AMPK) activation acts as a positive regulator of autophagy by inhibiting mammalian target of rapamycin (mTOR) in the state of intracellular energy deficiency [13]. Sirtuin 1 (SIRT1), a homologue of mammalian silencing information regulator 2, is an NAD^+^-dependent deacetylase that regulates protein activity by modifying the acetylation of molecules, transcription factors and enzymes. SIRT1 participates in the regulation of autophagy by affecting autophagy-related gene 3 (Atg3), Atg7 and microtubule-associated protein 1 (MAP1) light chain 3 (LC3) deacetylation. LC3, an initiator of autophagosomes, can be deacetylated by SIRT1 in the nucleus, which selectively activates LC3 and allows it to engage in autophagy [14, 15]. We previously reported that SIRT1 activation protected lipotoxicity-induced hepatic cell death by stimulating autophagy [16]. Akt (also known as protein kinase B, PKB), a key regulator of cellular survival, has also been confirmed to participate in the regulation of autophagy and lipotoxicity-induced hepatic cell death [17, 18].

There are no safe and effective clinical drugs to treat metabolic diseases so far. Accumulating evidence suggests that patients with abnormal liver metabolism can enhance their physical health indicators by adjusting their dietary structure, such as increasing the intake of fruits, vegetables, and whole grains [19]. Ferulic acid (FA), chemically known as 4-hydroxy-3-methoxy-cinnamic acid, is mainly cross-linked with cytoderm polysaccharides and lignin to form part of the cytoderm in plants. FA is widely found in the seeds of whole grains (bran, rice, wheat, etc.), vegetables (tomato, celery, spinach, etc.), and fruits (pineapple, grape, blackberry, etc.), with whole grains containing the highest FA content (up to 1000 mg/kg in rye) [20–22]. Accurate nutritional surveys about FA intake are lacking. Consumption of food source FAs can be estimated at a daily intake of approximately 150–200 mg [23]. Several biological functions of FA have been reported, such as antidiabetes, antioxidation, anti-inflammation and anticancer functions as well as blood lipid-lowering functions. FA is more easily absorbed by the body than other phenolic acids and stays in the blood for a longer amount of time [24]. FA acts as an antioxidant due to its scavenging of radicals, rather than the formation of phenoxyl radicals, as well as its inhibition of reactive oxygen species (ROS) generation through donation of a hydrogen atom. Previous studies showed that FA improved thioacetamide- and CCl4-induced hepatic fibrosis [25, 26] and diosbulbin B-, cadmium-, and streptozotocin-induced liver damage [27–29]. FA supplementation significantly improved hepatic lipid metabolism disorder and decreased liver injury in high-fat-diet-induced obese mice [30, 31]. However, few studies have been conducted to analyse the effect of FA on lipotoxicity-induced hepatic cell death, and the mechanisms are largely unclear.

The present study was designed to focus on the influence of FA on lipotoxicity-induced hepatocyte dysfunction, including apoptosis, mitochondrial dysfunction, and oxidative stress, and its potential molecular mechanisms. PA was chosen to establish a hepatic lipotoxicity model in vitro. We observed that incubation with FA markedly ameliorated PA-induced apoptosis, LDH leakage, mitochondrial membrane potential (MMP) reduction, ROS generation, and inflammatory activation. FA triggered SIRT1 upregulation, which in turn activated autophagy, which was mechanistically involved in the beneficial role of FA against lipotoxicity. Hence, our study reveals that FA is a potential effective phytochemical compound resistant to hepatic lipotoxicity.

## Material and methods

### Chemicals

All chemicals, including PA, bovine serum albumin (BSA), dimethylsulfoxide (DMSO), and chloroquine (CQ), were purchased from Sigma-Aldrich (St. Louis, MO). FA was provided by Chengdu Herbpurify Co., Ltd. (Sichuan, China). PA-BSA conjugates were prepared as described previously [16]. All experiments contained a control/vehicle group that was exposed to the same amount of solvent (e.g., BSA or DMSO).

### Cell culture

AML-12, a nontransformed mouse hepatocyte cell line, was obtained from the American Type Culture Collection (ATCC, Manassas, VA). Cells were cultured in a 1:1 mixture of Dulbecco’s Modified Eagle Medium/Ham’s Nutrient Mixture F-12 (DMEM/F-12, HyClone, Logan, Utah) containing 10% (v/v) foetal bovine serum, 10 μg/mL insulin, 5.5 μg/mL transferrin, 5 ng/mL sodium selenite, 40 ng/mL dexamethasone, 100 U/mL penicillin, and 100 μg/mL streptomycin, at 37 °C in a humidified O_2_/CO_2_ (19:1) atmosphere.

### Cell death assays

Cells were seeded into plates and allowed to grow to approximately 80% confluency and then incubated with the indicated treatments. Cell viability was detected by MTT assay, lactate dehydrogenase (LDH) release assay, and Hoechst staining. For the MTT assay, MTT (Solarbio, Beijing, China) was added to each well to a final concentration of 5 mg/mL and maintained at room temperature for 4 h. Then, DMSO was added and incubated on a plate shaker for 10 min. The absorption was detected at 470 nm using a microplate reader (Dynatech, El Paso, Texas, MR-4100). For the LDH assay, the medium was collected for LDH analysis using an LDH kit (Pierce, Rockford, IL) according to the manufacturer's instructions. The absorption values were measured at 340 nm using a microplate reader (Dynatech, MR-4100). Cell viability was also assessed by Hoechst 33,342 (Sigma-Aldrich) staining. The plate was washed twice with chilled phosphate-buffered saline (PBS) after staining for 30 min. The nuclear morphological changes were examined by fluorescence microscopy (Nikon, Tokyo, Japan, TE2000-U).

### ROS detection

ROS content was determined by a 2,7-dichlorodihydrofluorescein diacetate (DCFH-DA, Sigma-Aldrich) probe. DCFH-DA (10 μM final concentration) was added to each well and allow to stain the cells at room temperature for 30 min. Chilled PBS was used to wash the cells three times. The fluorescence intensity was measured with an inverted fluorescence microscope (Nikon, TE2000-U). ImageJ 1.51 software was used to quantify the mean fluorescence intensity (MFI) from five random fields.

### MMP assay

MMP was assessed by staining with the fluorescent dye rhodamine 123 (Rh123, Solarbio). Cells were stained with Rh123 (100 μg/mL final concentration) for 45 min at room temperature. Then, the cells were washed with chilled PBS to remove excess dye. The fluorescence intensity was measured with an inverted fluorescence microscope (Nikon, TE2000-U). ImageJ 1.51 software was used to analyse MFI from five random fields.

### RNA interference

Small interfering RNA (siRNA) targeting mouse SIRT1 was purchased from GenePharma Co., Ltd. (Shanghai, China). SiRNA-Mate (GenePharma) was utilized to deliver siRNA to the targeted cells according to the manufacturer’s protocol. Scrambled siRNA (GenePharma) was applied in the negative control group. Silencing efficiency was verified by quantitative real-time PCR and Western blot analysis.

### RNA extraction and quantitative real-time PCR

Intracellular total RNA was harvested by TRIzol (Invitrogen, Carlsbad, CA). qRT-PCR was performed with a StepOnePlus Real-Time PCR System (Applied Biosystems, Foster City, CA). The data were analysed by the 2^−(△△Ct)^ method. *Rn18s* was used as housekeeping gene for calibration. The primer sequences are listed in Table 1.

**Table 1:** List of primers

Gene	Forward primer (5′–3′)	Reverse primer (5′–3′)
*SIRT1* *IL-6*	GGAGCAGATTAGTAAGCGGCTTG CCGGAGAGGAGACTTCACAGC	GTTACTGCCACAGGAACTAGAGG AGAATTGCCATTGCACAAC
*IL-1beta*	TCCAGGATGAGGACATGAGCAC	GAACGTCACACACCAGCAGGTTA
*Rn18s*	GAATGGGGTTCAACGGGTTAA	GGTCTGTGATGCCCTTAGA

### Western blot analysis

Western blotting was performed as previously described [16]. The primary antibodies used were as follows: anti-cleaved-caspase-3, anti-Bcl-2, anti-Bax, anti-phosphorylated-AMPK, anti-AMPK, anti-phosphorylated-Akt, anti-Akt, anti-LC3B, anti-p62, anti-Beclin1, anti-ATG5, anti-ATG7, anti-Acetylation, anti-Lamin B and anti-SIRT1 antibody from Cell Signaling Technology, Inc. (Danvers, MA). The anti-β-tubulin1 antibody was from Boster Biological Technology (Wuhan, China). The secondary antibodies were provided by Boster Biological Technology.

### Analysis of autophagic flux

The autophagic flux was measured as previously described [32]. In brief, chloroquine (CQ) was added prior to FA treatment to inhibit lysosome acidification. Autophagic flux was determined by detecting GFP-LC3 puncta with a laser scanning confocal microscope (Zeiss, Jena, German, LSM880), and LC3 II expression was determined by Western blotting. For GFP-LC3 fluorescence detection, cells were transiently infected with recombinant GFP-LC3 lentivirus (GeneChem, Shanghai, China).

#### Statistical analysis

All experimental data are from at least three independent experiments and are shown as the mean ± SD. Statistical significance was determined by Student's t-test or one-way ANOVA followed by Tukey's multiple comparisons test. All tests were performed with SPSS 19.0. A value of *p* < 0.05 was considered significant.

## Results

### FA protects against hepatocyte cell death induced by PA

**Fig. 1:**
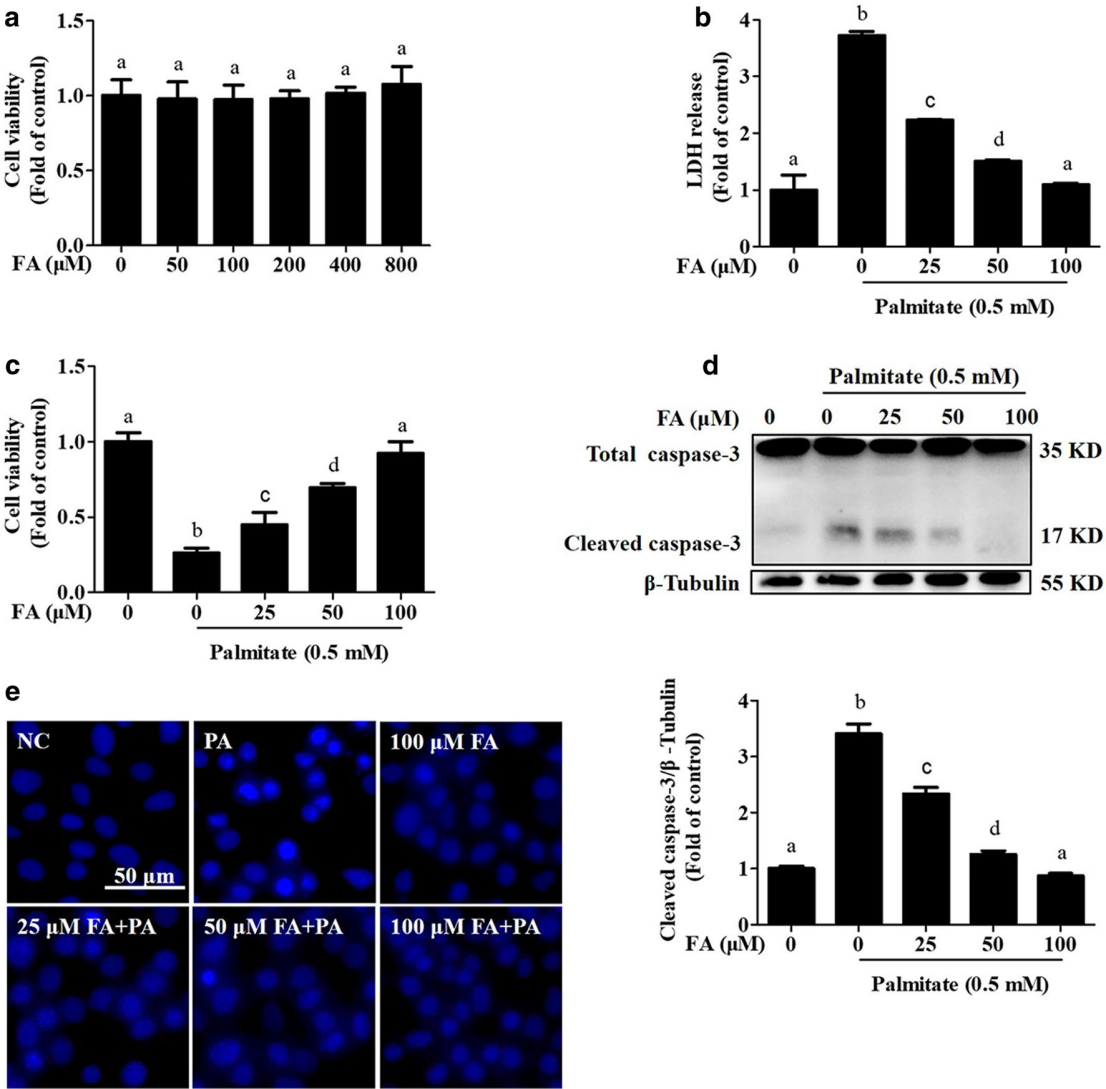
Ferulic acid protects hepatocytes against palmitate-induced cell death. **a** AML-12 cells were treated with different concentrations (0, 50, 100, 200, 400, and 800 μM) of ferulic acid (FA). After 24 h of incubation, cell viability was analysed by MTT assay. **b**, **c** AML-12 cells at 80% confluence were incubated with 0.5 mM palmitic acid (PA) for 12 h after pretreatment for 2 h with different concentrations (0, 25, 50, and 100 μM) of FA. Cell death was measured by LDH release in the culture medium and MTT assay. **d** Total cellular lysates were probed for cleaved-caspase-3 by immunoblotting. **e** The nuclear morphology was detected by Hoechst staining. All values are shown as the means ± SD from three or more independent batches of cells. Bars with different superscripts are significantly different at *p* ˂ 0.05

The cytotoxicity of FA was initially determined in AML-12 hepatocytes. No significant cytotoxicity was observed at doses up to 800 μM (Fig. 1a). Based on these results combined with those of previous studies [8, 33], incremental doses of FA (0, 25, 50, and 100 µM) were selected to evaluate the protective effect of FA against palmitate-induced lipotoxicity. The results indicated that FA significantly suppressed PA-induced hepatocyte cell death in a dose-dependent manner (Fig. 1b, c). FA exposure also prevented PA-induced caspase-3 cleavage, a typical indicator of apoptosis, with an optimal dose of 100 μM (Fig. 1d). In addition, PA-induced DNA condensation and nuclear fragmentation were also inhibited by FA pretreatment (Fig. 1e). To test the generality of our findings, we conducted similar experiments in a human hepatocyte cell line (HepG2). Our results showed that FA incubation markedly reversed PA-induced cell death in HepG2 cells (Additional file 1: Fig. S1). All these results suggested that FA exhibited a strong protective role against hepatic lipotoxicity induced by PA.

### FA improves lipotoxicity-induced mitochondrial dysfunction in hepatocytes

**Fig. 2:**
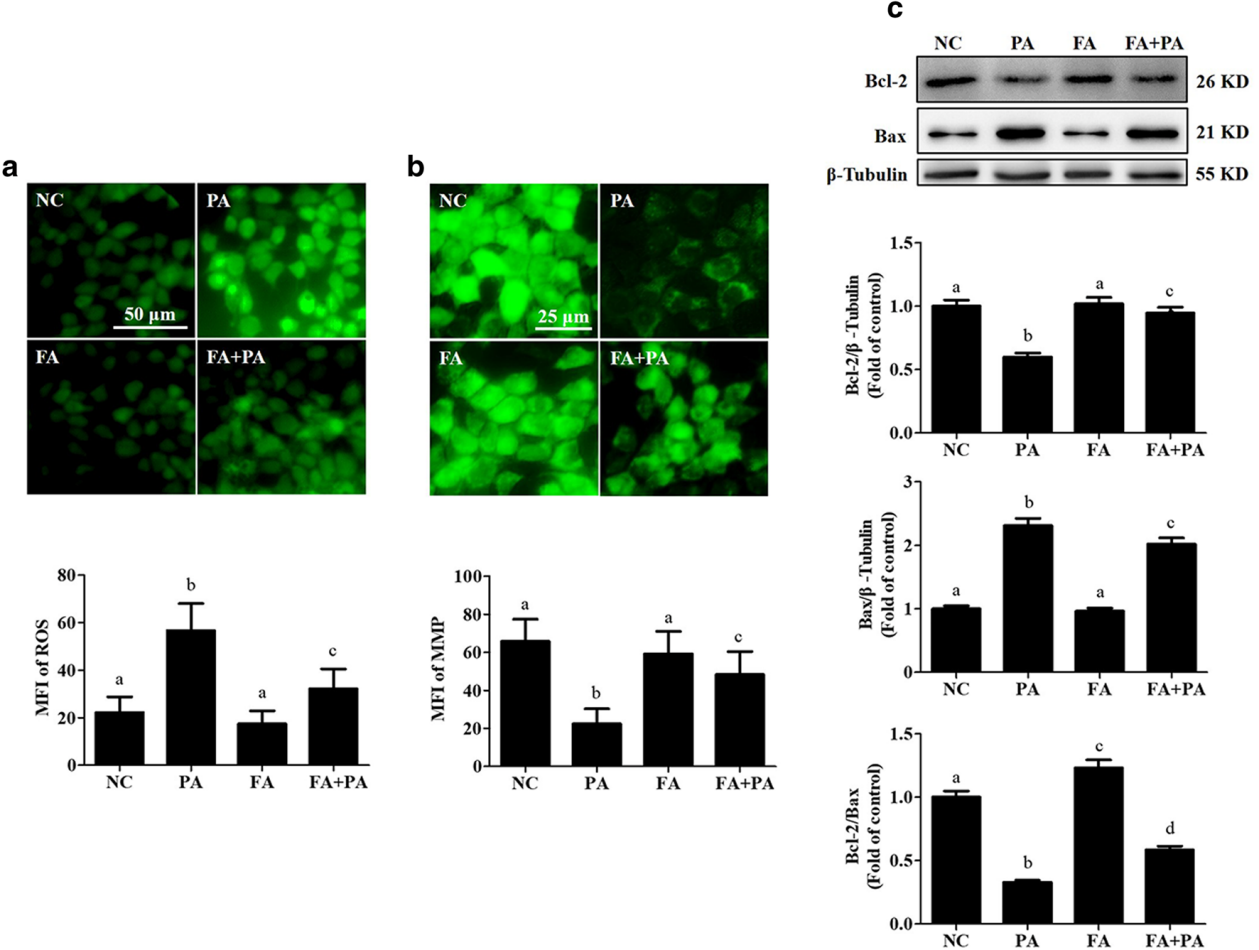
Ferulic acid improves lipotoxicity-induced mitochondrial dysfunction in hepatocytes. AML-12 cells were treated with 0.5 mM palmitic acid (PA) with or without 100 μM ferulic acid (FA) pretreatment for 2 h. **a** The intracellular reactive oxygen species (ROS) levels were analysed by DCFH-DA staining. **b** The mitochondrial membrane potential (MMP) was detected by Rh123 staining. **c** Bcl-2 and Bax were detected by immunoblotting. All values are shown as the means ± SD from three or more independent batches of cells. Bars with different superscripts are significantly different at *p* ˂ 0.05

In view of the strong antioxidant effect of FA, we subsequently tested the protective ability of FA on mitochondria, the main source of intracellular ROS. Our results showed that PA exposure significantly potentiated intracellular ROS levels, while FA pretreatment effectively prevented PA-triggered excessive ROS generation (Fig. 2a). Meanwhile, FA pretreatment also dramatically recovered the MMP suppressed by PA (Fig. 2b). Moreover, the alteration of apoptosis-related mitochondrial proteins, including Bcl-2 and Bax, was significantly reversed by FA pretreatment (Fig. 2c).

### Autophagy activation contributes to FA-mediated inhibition of lipotoxicity

**Fig. 3:**
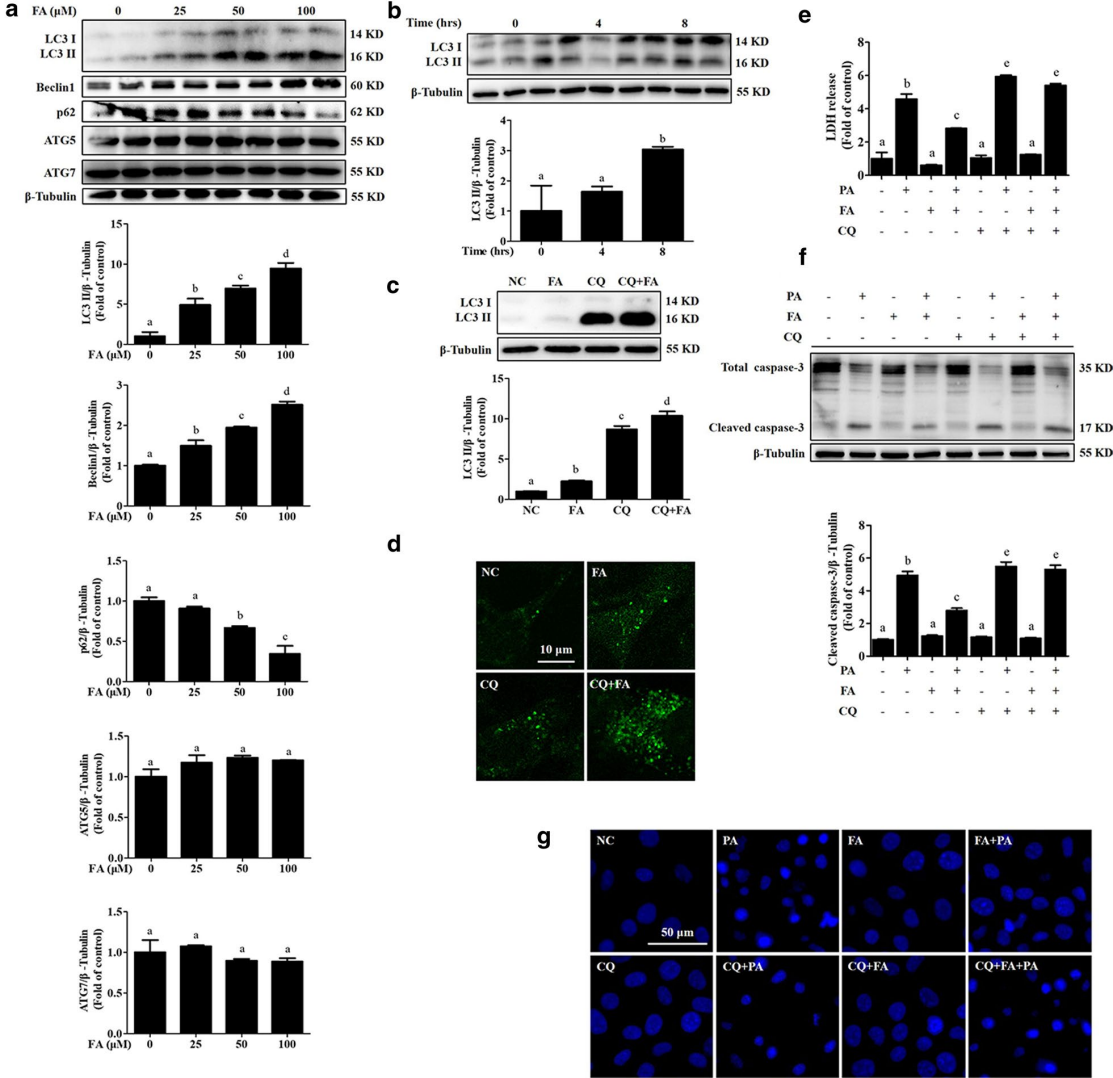
Autophagy activation contributes to ferulic acid-mediated protection against lipotoxicity. **a** AML-12 cells were treated with different concentrations (0, 25, 50, and 100 μM) of ferulic acid (FA) for 12 h. LC3, ATG5, ATG7, Beclin1, and p62 were detected by immunoblotting. **b** AML-12 cells were treated with 100 μM FA for 0, 4, or 8 h. LC3 was detected by immunoblotting. **c** AML-12 cells were pretreated with 20 μM chloroquine (CQ, a lysosomal acidification blocker) for 1 h with or without 100 μM FA exposure for 12 h. LC3 was detected by immunoblotting. **d** AML-12 cells were transfected with the mRFP-GFP-LC3 plasmid before chemical exposure. The LC3 puncta were detected by confocal microscopy after the indicated treatments. **e** Cells were pretreated with CQ for 1 h before FA exposure. FA (100 μM) was added 2 h before palmitic acid (PA) exposure. LDH release was measured. **f** Caspase-3 cleavage was detected. **g** Nuclear morphology was detected by Hoechst staining. All values are shown as the means ± SD from three or more independent batches of cells. Bars with different superscripts are significantly different at *p* ˂ 0.05

We previously reported that the activation of autophagy protects hepatocytes from PA-induced lipotoxicity [8]. In this study, we found that FA exposure significantly stimulated hepatic autophagy in AML-12 cells, as evidenced by the observation of increased Beclin1 expression and LC3 II conversion, both of which are well-established markers of autophagy induction, and reduced expression of p62, a protein specifically degraded by autophagy, induced by FA in a dose-dependent and time-dependent manner (Fig. 3a, b). Although we did not observe an upregulatory effect of FA on the expression of other autophagy-related genes, including ATG5 and ATG7 (Fig. 3a), FA incubation markedly stimulated autophagic flux, which was confirmed by increased LC3 II conversion and LC-3 puncta formation in the presence of chloroquine (Fig. 3c, d). Importantly, the protective effect of FA against lipotoxicity was robustly blocked by autophagy inhibition (Fig. 3e–g), indicating that autophagy activation participated in the beneficial effect of FA against lipotoxicity.

### SIRT1 upregulation participates in FA-induced autophagy

**Fig. 4:**
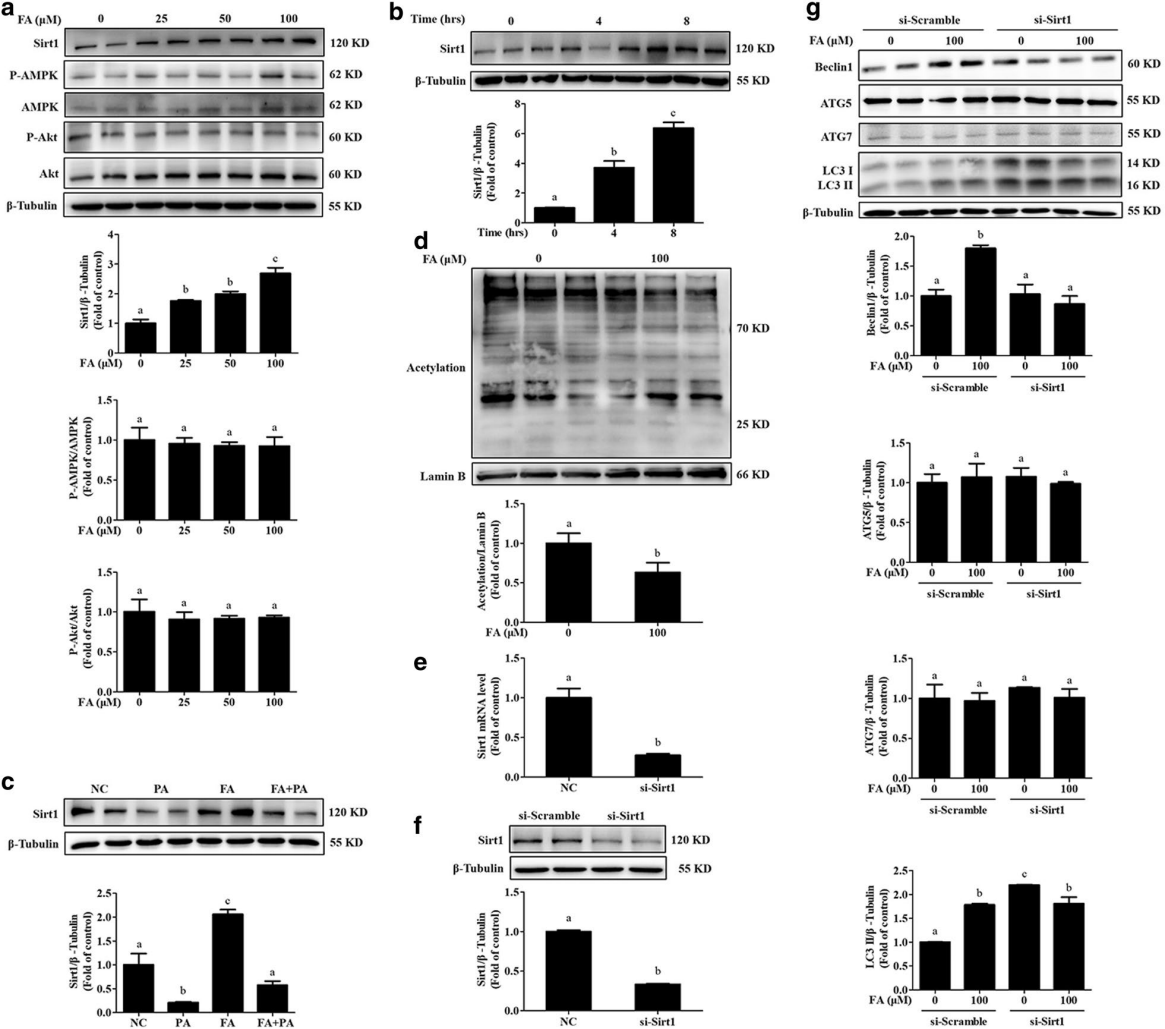
SIRT1 upregulation participated in ferulic acid-induced autophagy. **a** AML-12 cells were treated with different concentrations (0, 25, 50, and 100 μM) of ferulic acid (FA) for 12 h. Total cell lysates were probed by immunoblotting for SIRT1, phosphorylated AMPK, and phosphorylated Akt. **b** Cells were treated with 100 μM FA for 0, 4, and 8 h. SIRT1 protein abundance was detected. **c** Nuclear protein acetylation was detected after FA (100 μM) treatment for 12 h. **d** FA (100 μM) was added 2 h before 0.5 mM palmitic acid (PA) exposure. After 12 h of incubation, SIRT1 was detected by immunoblotting. **e**, **f** AML-12 cells were transfected with si-SIRT1 or scramble siRNA. The silencing efficiency was verified by SIRT1 mRNA and protein expression. **g** Autophagy-related proteins, including ATG5, ATG7, Beclin1 and LC3, were detected by immunoblotting. All values are shown as the means ± SD from three or more independent batches of cells. Bars with different superscripts are significantly different at *p* ˂ 0.05

Subsequently, we investigated the upstream regulators by which FA stimulated autophagy. Previous studies have reported that FA treatment promotes the phosphorylation of both AMPK and Akt [34, 35], whose induction is mechanistically involved in the activation of autophagy [13, 18]. Therefore, we asked whether the activation of these two kinases contributes to FA-regulated autophagy in hepatocytes. Unexpectedly, our data showed that FA treatment did not increase the expression of phosphorylated AMPK or Akt (Fig. 4a). However, we observed that SIRT1, which is another upstream regulator of autophagy [36], was upregulated by FA treatment in a dose-dependent and time-dependent manner (Fig. 4a, b). We also tested the activity of SIRT1 in response to FA treatment by detecting the nuclear protein acetylation status. Our data showed that FA exposure significantly stimulated the activity of SIRT1 based on the observation of less acetylated protein in the hepatocyte nuclei (Fig. 4c). PA-mediated suppression of SIRT1 protein expression was obviously reversed by FA incubation (Fig. 4d). Importantly, FA treatment failed to stimulate autophagy in SIRT1-silenced hepatocytes (Fig. 4e–g), indicating that SIRT1 participated in FA-stimulated autophagy. We also observed a higher level of LC3 II in SIRT1-knockdown cells (Fig. 4g), probably because SIRT1 deficiency impeded autophagic flux and hence blocked LC3 II degradation. To confirm this possibility, we measured the expression of p62, which can be specifically degraded by autophagy. In support of our hypothesis, and in line with the results of a previous study [14], p62 obviously accumulated in SIRT1-silenced cells (Additional file 1: Fig. S2).

### SIRT1-regulated autophagy is mechanistically involved in the FA-mediated alleviation of lipotoxicity

**Fig. 5:**
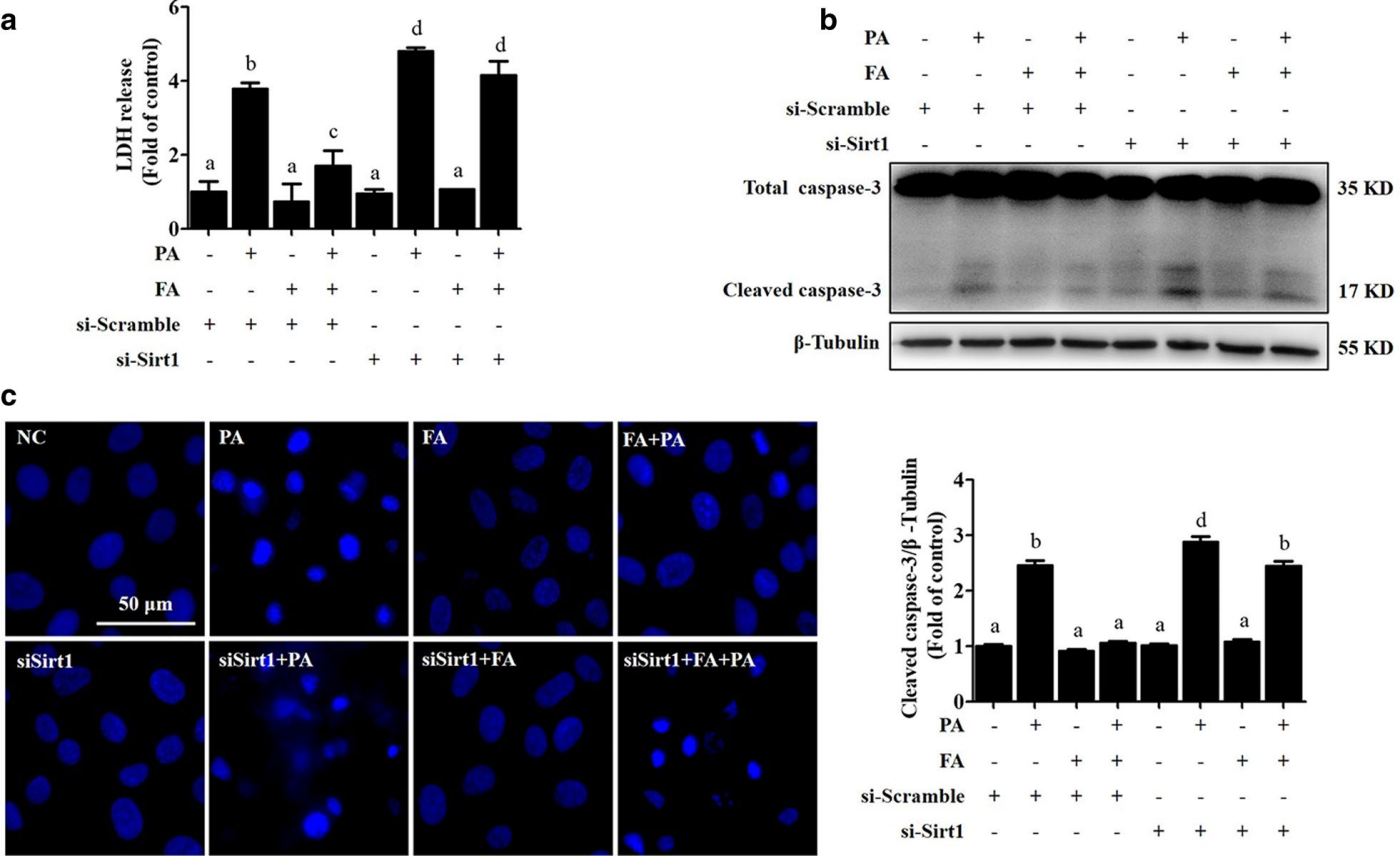
SIRT1-regulated autophagy is mechanistically involved in ferulic acid-alleviated lipotoxicity. AML-12 cells were transfected with si-SIRT1 or scramble siRNA before ferulic acid (FA) exposure. FA (100 μM) was added 2 h before 0.5 mM palmitic acid (PA) exposure (12 h). **a**, **b** LDH release and caspase-3 cleavage were detected. **c** Nuclear morphology was observed by Hoechst staining. All values are shown as the means ± SD from three or more independent batches of cells. Bars with different superscripts are significantly different at *p* ˂ 0.05

We then analysed the participation of SIRT1-mediated autophagy in the protective effect of FA against lipotoxicity in AML-12 hepatocytes. The results showed that genetically knocking down SIRT1 significantly blocked the protective effect of FA against lipotoxicity induced by PA treatment, as evidenced by the detection of LDH release, cleaved-caspase-3 expression, and nuclear staining (Fig. 5a–c).

### FA improves PA-induced proinflammatory cytokine activation in hepatocytes

**Fig. 6:**
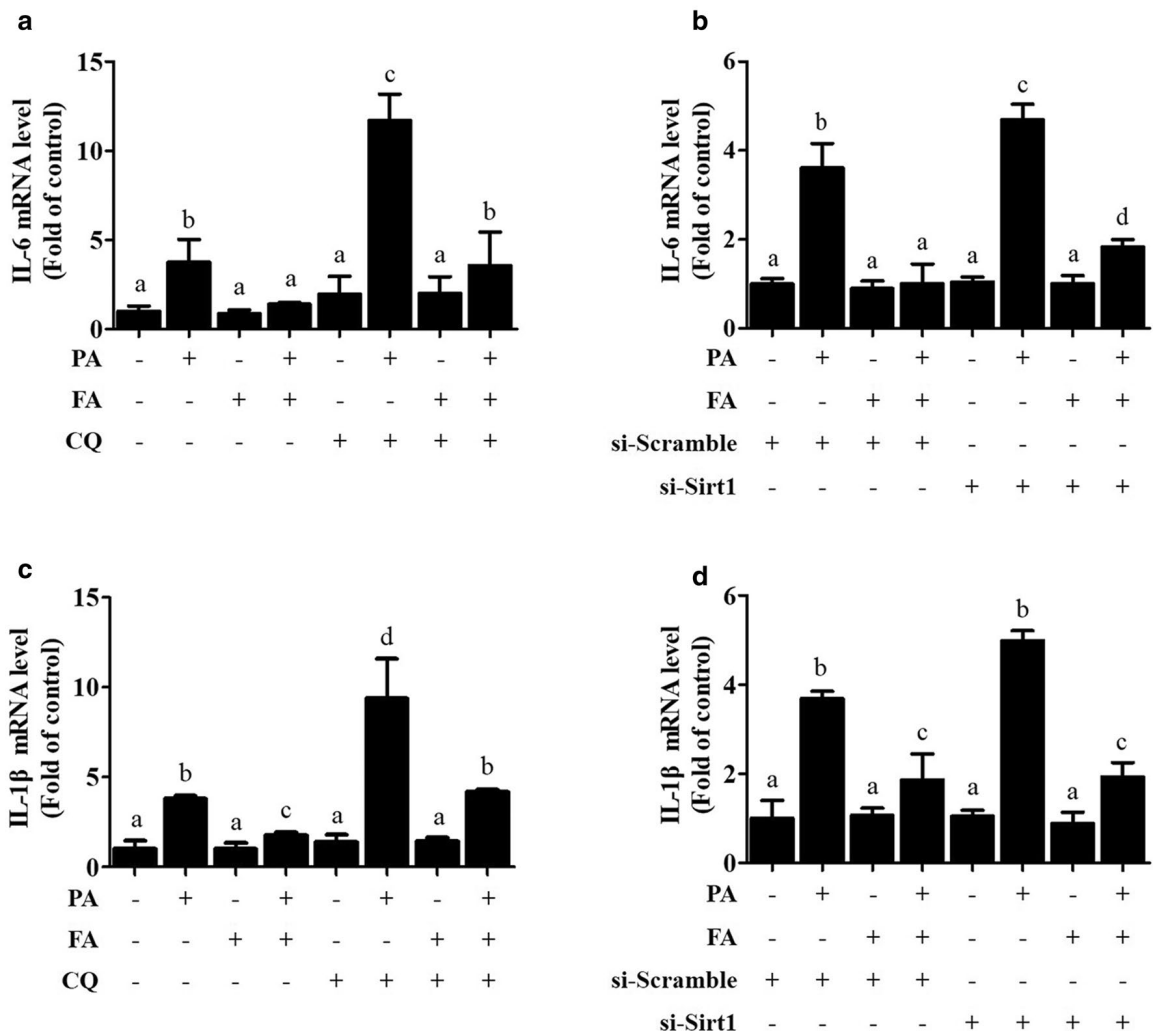
Ferulic acid improves PA-induced proinflammatory cytokines activation in hepatocytes. AML-12 cells were pretreated with or without ferulic acid (FA, 100 μM) for 2 h before 12 h of palmitic acid (PA, 0.5 mM) exposure. Chloroquine (CQ, 20 μM) was added 1 h before FA exposure. For SIRT1 silencing, treatments were carried out after transfecting cells with si-SIRT1 or scramble siRNA. Gene expression of proinflammatory factors was analysed by quantitative real-time PCR. **a**, **b** *IL-6* mRNA. **c**, **d** *IL-1β* mRNA. All values are shown as the means ± SD from three or more independent batches of cells. Bars with different superscripts are significantly different at *p* ˂ 0.05

The anti-inflammatory role of FA was investigated in this study. Our results showed that PA exposure significantly transcriptionally stimulated proinflammatory factors, including IL-1beta and IL-6, while FA pretreatment markedly reversed PA-induced inflammatory reactions (Fig. 6a–d). We further asked that whether SIRT1/autophagy pathway was mechanistically involved in FA-inhibited inflammation. Our data showed that neither inhibiting autophagy by CQ nor silencing SIRT1 by special siRNA blocked FA-protected IL-1beta and IL-6 expressions (Fig. 6a–d), which indicated that a SIRT1/autophagy independent pathway might contribute to the anti-inflammatory effect of FA in hepatocytes.

## Discussion

In this study, we identified that FA, a nontoxic phenolic acid, exerted a strong antiapoptotic role in PA-induced hepatic lipotoxicity. FA intervention significantly alleviated lipotoxicity-induced mitochondrial dysfunction and inflammation in AML-12 hepatocytes. Our data suggested that the SIRT1-mediated autophagy signalling pathway contributed to the beneficial effects of FA mentioned above.

Lipotoxicity plays an essential pathological role in the development of several metabolic diseases [37]. In the liver, excessive FFAs may initiate hepatocyte injury, inflammation, and even apoptosis, which in turn leads to liver dysfunction and promotes the occurrence of various metabolic diseases [38, 39]. It is commonly recognized that the major detrimental role of lipotoxicity is not caused by neutral triglyceride deposition but rather originates from excessive free SFAs, among which PA is the commonly used lipotoxicity-inducing compound [5, 7, 40]. Accumulated evidence has proven that strategies for alleviating hepatic lipotoxicity effectively improve metabolic diseases, such as NAFLD [41]. To date, no safe and efficient clinical drug to prevent lipotoxicity has been officially approved. Many epidemiological studies have reported that improving dietary habits, especially increasing the intake of plant-based foods, such as whole-grain food, was beneficial for the prevention of hepatic metabolic disorders in NAFLD patients [42]. Therefore, phytochemicals extracted from plant foods or medical herbs provide a feasible alternative for the treatment of lipotoxicity-related metabolic diseases. FA, which is widely present in whole grains and is found at a concentration of 1000 mg/kg in rye [22], was recently reported to improve high-fat-diet-induced hepatic metabolic disorder in experimental animals [30, 31, 43]. Several studies have demonstrated that FA is a strong scavenger of excessive ROS, and ROS induction is mechanistically involved in lipotoxicity-induced apoptosis [44, 45]. However, limited studies have been conducted to investigate the protective role of FA against lipotoxicity-induced cell death in hepatocytes. In this study, we reported for the first time that FA intervention significantly alleviated PA-induced apoptosis in hepatocytes, which was confirmed by the detection of LDH release, caspase-3 cleavage, and nuclear morphology.

We subsequently analysed the potential mechanisms behind the protective effects of FA. Autophagy is a conserved and complex quality control pathway that plays a crucial role in eliminating damaged proteins and organelles [46]. Upon autophagy induction, LC3, a mammalian homologue of Atg8, controls the formation of autophagosomes and lysosomes as well as the degradation of substrates. LC3 I is regulated by phosphatidylethanolamine at its glycine residue to become LC3 II, which is bound to both the outer and inner membranes of the autophagosome [47]. Beclin1 (also termed BECN1), a homologue of yeast Atg6, not only participates in the formation of autophagosomes but also regulates autophagic activity [48]. SQSTM1 (sequestosome 1, also known as p62) is a selective autophagy receptor that transports ubiquitinated targets to autophagosomes [49]. It has been well documented that autophagic flux is impaired in the livers of individuals with metabolic diseases, such as NAFLD [50–52]. The activation of autophagy improved hepatic metabolic disorders by removing excess lipid droplets from hepatocytes and alleviated liver injury [53]. We previously reported that autophagy activation protected hepatocytes from SFA-induced hepatic apoptosis [8]. Therefore, we hypothesized that autophagy activation contributed to the beneficial role of FA. In support of our hypothesis, FA treatment markedly stimulated autophagy in hepatocytes based on the observations of increased autophagic flux, enhanced Beclin1 expression and LC3 II conversion, and reduced p62 expression. In line with our observations, FA has also been reported to stimulate autophagy in other types of cells, including renal cells, brain microvascular endothelial cells, and myocardial cells [54–56]. Importantly, inhibition of autophagy significantly blocked the protective role of FA against PA-induced apoptosis, indicating that autophagy induction was mechanistically involved in the FA-mediated alleviation of lipotoxicity in hepatocytes.

Lipotoxicity-induced mitochondrial dysfunction, excessive ROS production, and even programmed apoptosis play critical roles in the pathological process in hepatic metabolic diseases [4]. Selective degradation of damaged mitochondria by autophagy, also termed mitophagy, helps to maintain the integrity of cell function. Recent evidence has shown that the induction of mitophagy prevents high-fat-diet-induced liver injury [57, 58]. In addition, PA decreased mitophagy, leading to mitochondrial dysfunction characterized by extensive mito-ROS production and MMP loss [59]. Data from our study clearly revealed that FA treatment significantly abrogated PA-induced mitochondrial dysfunction by improving MMP and intracellular ROS levels. However, more direct evidence on mitophagy in FA-treated hepatocytes is still needed in future studies.

Several mechanisms have been identified in the regulation of autophagy. SIRT1, an NAD^+^-dependent deacetylase, regulates autophagy initiation by mediating LC3 deacetylation [36]. SIRT1 activation exerted a positive effect on the regulation of liver lipid metabolism, oxidation and inflammation [60], whereas SIRT1 depletion accelerated hepatic injury in the pathogenesis of NAFLD [60, 61]. We recently reported that SIRT1 induction has a protective effect against PA-induced hepatocellular death [16]. This evidence prompted us to hypothesize that SIRT1-regulated autophagy contributes to FA-mediated protection against lipotoxicity. This notion was supported by the following evidence: First, FA treatment obviously stimulated SIRT1 expression in a dose-dependent and time-dependent manner. Similar effects were also observed in FA-treated skeletal muscle cells, bone, and testis [62–64]. Second, genetically knocking down SIRT1 robustly abolished FA-stimulated autophagy induction. Last but most importantly, FA-mediated protection against lipotoxicity was robustly blocked in SIRT1-silenced hepatocytes. We also detected the potential targets through which SIRT1 regulated autophagy. Previous studies have reported that SIRT1 activation stimulated Beclin1, Atg5, and Atg7 upregulation in virous types of cells [65–67]. In this study, our data clearly indicated that Beclin1 induction but not Atg5 and Atg7 contributed to SIRT1-stimulated autophagy in the presence of FA, evidenced by the observations that FA treatment only enhanced the expression of Beclin1 without affecting Atg5 and Atg7, and this enhancement was blocked by SIRT1 silencing, which was supported by a previous study that autophagy could be stimulated independently of Atg5 and Atg7 [68–70]. It has also been reported that FA stimulated autophagy via Beclin1 involved pathway in the penumbral cortex of rats [71]. The above evidence implied that Beclin1 was a potential target of SIRT1 in FA-stimulated autophagy in hepatocytes. An interesting question will be whether and how SIRT1 regulates the acetylation level of autophagy-related genes in the presence of FA, which should be investigated in the future study. Additionally, AMPK, a central sensor of intracellular energy, is a key regulator of autophagy by inhibiting the downstream target mTOR complex 1, which is a negative regulator of autophagy. Several studies have reported the reciprocal regulatory relationship between SIRT1 and AMPK [72]. The activation of AMPK significantly eliminated lipotoxicity-induced hepatocyte death [73]. FA was shown to activate AMPK in skeletal muscle cells and cardiac myocytes [34, 60]. We therefore analysed the involvement of phosphorylated AMPK in FA-treated hepatocytes. Unexpectedly, FA incubation did not activate AMPK phosphorylation in AML-12 hepatocytes, which excluded the participation of AMPK in FA-mediated protection against lipotoxicity. The phosphatidylinositol 3-kinase (PI3K)/Akt pathway, whose activation promotes cell survival under lipotoxic conditions [17], is mechanistically involved in autophagy induction [18]. Reports on the regulation of PI3K/Akt by FA have been inconsistent. PI3K/Akt was inhibited by FA in tumour cells [74] but stimulated during cellular dysfunction induced by detrimental stimuli [75]. In this study, we observed that Akt phosphorylation was not significantly regulated by FA treatment in AML-12 hepatocytes, implying that the PI3K/Akt pathway did not participate in FA-induced autophagy or in other beneficial effects of FA against lipotoxicity.

Proinflammatory factor-triggered cytotoxicity plays a detrimental role in the development of hepatic metabolic disorders. Accumulating studies, including ours, have indicated that PA exposure transcriptionally stimulates the expression of proinflammatory factors, including IL-1beta and IL-6 [76–78]. In the present study, we observed that FA intervention markedly reversed PA-induced activation of proinflammatory factors in hepatocytes, which was in line with the fact that FA inhibited proinflammatory reactions in other types of cells [79, 80]. However, our data showed that SIRT1-regulated autophagy pathway was not involved in FA-inhibited inflammation. More mechanistical studies are still needed to clarify on how does FA regulate lipoxicity-induced inflammation in hepatocytes. In view of the crosstalk between oxidative stress and inflammation, we cannot say whether FA inhibited lipotoxicity by acting on the inflammatory signaling pathway or by improving oxidative stress; this question will need further investigation.

## Conclusions

In summary, our study reported that by activating the SIRT1/autophagy pathway, FA treatment protects hepatocytes against lipotoxicity-induced apoptosis. Our findings provide a new mechanism that may advance our understanding of the biological value of FA in hepatic metabolic diseases. This study highlights the potential value of FA as a dietary supplement in preventing and/or treating liver diseases with lipotoxicity as a typical pathological feature.

## Supplementary Information

**Additional file 1**. Supplementary data file.

## Data Availability

Not applicable.

## Abbreviations

FA: Ferulic acid
NAFLD: Non-alcoholic fatty liver disease
FFAs: Free fatty acids
SFAs: Saturated fatty acids
USFAs: Unsaturated fatty acids
PA: Palmitate acid
AMPK: Adenosine monophosphate-activated protein kinase
mTOR: Mammalian target of rapamycin
SIRT1: Sirtuin 1
Atg: Autophagy-related genes
LC3: Microtubule-associated protein 1 light chain 3
PKB: Protein kinase B
SQSTM1 (also known as p62): Sequestrome1
MMP: Mitochondrial membrane potential
ROS: Reactive oxygen species
CQ: Choloroquine
LDH: Lactate dehydrogenase
DCFH-DA: 2,7-Dichlorodi-hydrofluorescein diacetate
Rh123: Rhodamine 123
