# Supplementary material for: Ferulic acid alleviates lipotoxicity-induced hepatocellular death through the SIRT1-regulated autophagy pathway and independently of AMPK and Akt in AML-12 hepatocytes
Source: Nutr Metab (Lond). 2021 Jan 19;18:13. doi: 10.1186/s12986-021-00540-9 (PMC7814733; doi:10.1186/s12986-021-00540-9)
Supplement: Supplementary file 1 — Additional file 1. Supplementary data file. [file 12986_2021_540_MOESM1_ESM.docx]

Supplementary Data


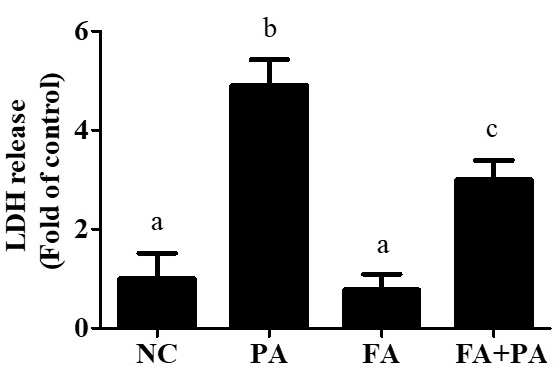


Figure S1 Ferulic acid alleviates palmitate-induced cell death in HepG2 cells. The human hepatoma cell line (HepG2) was obtained from Shanghai Institute of Cell Bank (Shanghai, China). Cells were cultured in Dulbecco’s Modified Eagle Medium (DMEM, Thermo Scientific Inc, VA) containing 10% (v/v) foetal bovine serum (FBS, Biological Industries, Israel), 100 U /mL penicillin and streptomycin (Thermo Scientific Inc, VA) at 37°C in a humidified O2/CO2 (19:1) atmosphere. Cells at 80% confluence were incubated with 0.5 mM palmitic acid (PA) for 12 h after pretreatment for 2 h with 100 μM FA. LDH release was detected. All values are shown as the means ± SD from three or more independent batches of cells. Bars with different superscripts are significantly different at *p* ˂ 0.05.


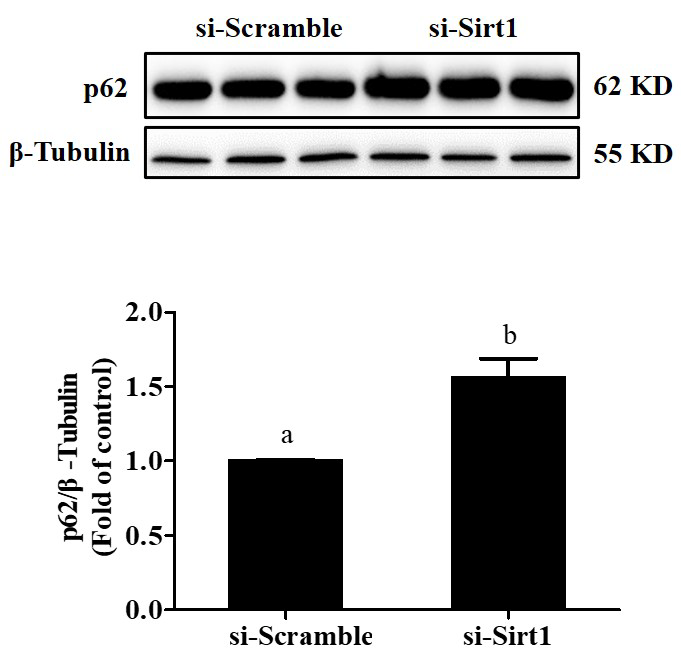


Figure S2 SIRT1-knockdown increases p62 expression in hepatocytes. AML-12 cells were transfected with si-SIRT1 or scramble siRNA. p62 protein expression was detected by immunoblotting. All values are shown as the means ± SD from three or more independent batches of cells. Bars with different superscripts are significantly different at *p* ˂ 0.05.
